# Job Accommodations and Job Loss From Unilateral Vocal Fold Paralysis

**DOI:** 10.1002/ohn.1334

**Published:** 2025-06-12

**Authors:** Koffi L. Lakpa, Andrew Bowen, Ezra Menon, Sydney Ring, Peter Nordby, Miranda Rasmussen, Natalia Arroyo, Jiwei Zhao, Sara Fernandes‐Taylor, David O. Francis, CoPE Collaborative

**Affiliations:** ^1^ University of Wisconsin School of Medicine and Public Health Madison Wisconsin USA; ^2^ University of Wisconsin School of Medicine and Public Health, Division of Otolaryngology–Head and Neck Surgery Madison Wisconsin USA; ^3^ Wisconsin Surgical Outcomes Research Program (WiSOR), Department of Surgery School of Medicine and Public Health University of Wisconsin‐Madison Madison Wisconsin USA; ^4^ University of Wisconsin School of Medicine and Public Health, Carbone Cancer Center Madison Wisconsin USA; ^5^ Department of Biostatistics and Medical Informatics University of Wisconsin School of Medicine and Public Health Madison Wisconsin USA

**Keywords:** accommodations, job loss, unilateral vocal fold paralysis

## Abstract

**Objective:**

Unilateral vocal fold paralysis (UVFP) is a debilitating injury that affects a person's ability to communicate and swallow. Although clinical aspects are understood, how UVFP affects a person's employment and job security is poorly characterized. This study aims to elucidate how frequently patients request job accommodations or experience job loss related to UVFP.

**Study Design:**

Cross‐sectional survey.

**Setting:**

Thirty‐four US tertiary care voice centers.

**Methods:**

We analyzed data from a prospective cohort of patients with UVFP recruited from 34 vocal Cord Paralysis Experience (CoPE) collaborative voice centers. Sociodemographic information, clinical and treatment history, and patient‐reported outcome measures were collected. Patients also provided employment information, whether they requested job accommodations, and if they experienced job loss due to UVFP.

**Results:**

In all, 613 participants (mean age 58 years, 65% women, 84% Caucasian) enrolled in CoPE. In total, 64 (10%) had requested job accommodations, and 46 (7.5%) reported job loss due to UVFP. Women (odds ratio [OR] 2.6, 95% CI: 1.4‐5.0, *P* = .004) and participants with higher levels of education were more likely to request work accommodations (OR 4.5, 95% CI: 1.4‐14.7, *P* = .01).

**Conclusion:**

UVFP has major impacts on employment with 1 in 10 patients requesting job accommodations and 1 in 13 experiencing job loss. Our results raise awareness of the importance of timely, effective treatment for UVFP and should alert treating clinicians to provide support to patients who may require job accommodations.

Unilateral vocal fold paralysis (UVFP), caused by injury to one recurrent laryngeal nerve, is a common and life‐changing complication of an estimated 500,000 head, neck, and chest surgeries performed annually in the United States.^1^ Altogether, it is estimated that 20,000 patients in the United States are diagnosed with UVFP each year including up to 1 in 10 patients undergoing thyroidectomy.^2^, ^3^ UVFP causes glottal incompetence, which means patients cannot close their vocal folds to produce a normal voice and fully protect their airway during swallowing.^4^, ^5^, ^6^ Quality of life sequelae from UVFP are significant; however, less well understood are the effects that this injury has on employment.

In the United States, 25% to 30% of the population rely on their voice as the primary tool for their occupation.^7^ The disability of an impaired voice has major ramifications on one's work productivity and effectiveness and may require job accommodations or even result in loss of employment. The Americans with Disabilities Act Amendments Act (ADAAA) of 2008 recognizes “speaking and communicating” disorders, prohibits discrimination, and ensures equal opportunity for persons with such disabilities, including making accommodations for communicative disability.^8^

Researchers have made efforts to understand how a patient's voice affects his or her job status.^9^, ^10^, ^11^ However, to our knowledge, only two single‐institution case series have evaluated the vocational impact of UVFP. One study found that 65% of those with UVFP reported moderate to extremely adverse effects on their future career options, and approximately 30% changed jobs.^12^ The other study reported that 27% of patients with UVFP required employment change even after undergoing definitive type I thyroplasty to correct their glottal insufficiency.^13^ Neither study evaluated the need for job accommodations or which occupations were most affected by the UVFP‐related communicative disability.

UVFP has major impacts on job productivity and employment status, but it is less clear what the magnitude is and which occupations are most affected. We attempt to address this knowledge gap by leveraging the vocal Cord Paralysis Experience (CoPE) Collaborative, composed of 34 US tertiary care centers who prospectively collect data on patients with UVFP,^14^ to estimate the US population‐level impact of UVFP on job accommodations and job loss.

## Methods

Details of the study protocol, inclusion criteria, study design, item development, cognitive interviews, and conceptual model have been published.^4^, ^5^, ^14^ Consequently, the methods are described in brief and focus on those relevant to the occupational impact of UVFP. The study was approved by the Committee for the Protection of Human Subjects at the University of Wisconsin (IRB No. 2017‐1259).

### Patient Population

To ensure a representative, national cohort of patients with UVFP, we formed the CoPE Collaborative, composed of 34 US tertiary care institutions and does not include VA medical centers (Supplemental Table S2, available online). Patients with UVFP were enrolled regardless of treatment status or time since diagnosis. Patients were excluded if they had bilateral vocal fold movement abnormalities, chronic voice disorders, dysphagia, or pulmonary disease that predated UVFP onset.

### Data Collection

Patients were electronically consented to on enrollment before completing the baseline survey. Patients' survey responses were captured using a Health Insurance Portability and Accountability Act (HIPAA)‐compliant online portal from April 2019 to April 2021. At baseline, participants completed items related to the CoPE patient‐reported outcome measure (PROM) development,^14^ a comorbidity questionnaire modified from the Behavioral Risk Factor Surveillance System,^15^ a UVFP disease questionnaire, and several existing PROMs that measure communication, eating, and quality of life.^16^, ^17^, ^18^ After baseline data collection, participants completed the disease questionnaire and PROM items three times (every 2 months).

Baseline surveys inquired about participants' demographics, diagnosis, symptoms, treatment, type of work, work status, and whether they requested job accommodations due to their UVFP. A “job accommodation” is defined as an adjustment to a job or work environment that makes it possible for an individual with a disability to perform their job duties.^19^ Participants were also asked if they had ever left a job due to their UVFP. Although accommodations can be requested for voice disorders,^8^ the specific types of work accommodations requested by the CoPE cohort were not queried.

### Survey Questions About Accommodations and Employment

For current employment, the survey asked, “Currently, are you doing any work for pay?” Those who responded that they were not working for pay were asked, “Which one of the following best describes the primary reason you are not currently working for pay?” The options included: retired, volunteering, not employed due to physical or mental health issues, laid off, quit a job to seek other employment, family responsibilities, and chosen not to be employed. Individuals had the option to respond that they are currently retired but have a history of requesting accommodations or experiencing job loss in the past.

The survey queried participants about their occupation by asking, “What kind of work do you do, or what is your main occupation? For example: accounts manager, teacher, waiter, or computer programmer.” Participants who listed a current occupation were categorized into 1 of 22 major groups using the Office of Management and Budget Standard Occupational Classification (SOC) system, using a previously published method^20^ (Supplemental Table S1, available online).

### Primary Outcomes

The focus of this investigation was the impact of UVFP on employment. The two primary outcomes were whether participants had (1) requested job accommodations from their employer (yes/no) and (2) experienced job loss, voluntarily or involuntarily (yes/no), due to UVFP.

### Variables

Variables collected in the survey included demographic data, educational attainment, income level, current employment status (yes/no), reasons why participants were not currently employed, whether participants had ever requested job accommodations (yes/no), most recent occupation, and whether they had ever left a job due to their UVFP (yes/no).

### Statistical Analyses

Baseline characteristics were reported as means for continuous variables and proportions for categorical variables. Logistic regression was used to determine associations between UVFP‐related accommodation requests and job loss with selected variables. Confounding by race, sex, education, and income was addressed by calculating the percent difference in the coefficients between the crude and adjusted models when accounting for the possible confounder. Effect modification based on age, sex, race, and education was evaluated by examining the statistical significance of interaction terms between the possible effect modifier and the outcome of interest in the logistic regression models.

Demographic variables were added to the model based on *t* test, chi‐square, or Fisher exact testing with a *P*‐value < .25, to determine any independent association with either UVFP‐related job departure or UVFP‐related accommodations. The threshold of *P* < .25 was chosen as it is commonly used in exploratory regression modeling in epidemiology.^21^, ^22^ This approach helps to ensure potentially important variables are not excluded prematurely during the initial stages of model building. Fisher's exact testing was used when the count of participants for a particular variable was less than five. Logistic regression models for each outcome variable were constructed in a top‐down fashion, beginning with a model containing all variables with a *P*‐value < .25. Subsequent models had variables with *P*‐values > .05 removed in a singular fashion until the final model was reached.

All models were executed using the “proc logistic” command in SAS version 9.4 to determine the impact each statistically significant variable had on the odds of having either UVFP‐related employment loss or requested UVFP‐related accommodations. We hypothesized a priori that accommodations were associated with job loss; therefore, accommodation was included in the analysis of factors related to job loss. Log odds were converted to odds ratios (ORs) and then converted to probabilities. Hosmer and Lemeshow goodness‐of‐fit tests were done showing adequate fit for the job departure and accommodation models: chi‐square 0.17 (*P* = .68) and 0.78 (*P* = .68), respectively. Testing for interaction yielded no statistically significant interaction terms. Additionally, testing for confounding found no difference in coefficients that differed greater than 10% between the crude and final models.

## Results

### Participant Demographics

A total of 613 participants completed CoPE questionnaires (white 84%, women 65%, older than 61 years: 50%; Table 1). Nearly a quarter of participants completed some college (21%) or a 4‐year college degree (25%). In all, 40% of respondents reported being retired at the time of the survey. The most common occupations were sales (16%), healthcare (14.1%), education (11.5%), and management (11.5%) (Supplemental Table S1, available online).

**Table 1:** Demographic Information of Cord Paralysis Experience Participants (n = 613)

Patient demographics	No. (%)
Sex	
Women	396 (64.6)
Men	214 (34)
Preferred not to say/self‐describe	3 (0.4)
Age	
18‐40‐year‐old	94 (15.3)
41‐60‐year‐old	211 (34.4)
61+‐year‐old	308 (50.2)
Race	
White	507 (83.7)
Asian	26 (4.2)
Black	48 (7.8)
Native Hawaiian or other Pacific Islander	2 (0.32)
American Indian or Alaskan	4 (0.64)
Other or unspecified	11 (1.79)
Ethnicity	
Hispanic	43 (7.03)
Education	
Some high school	15 (2.44)
High school or GED	103 (16.8)
Trade school	22 (3.59)
Some college	126 (20.6)
Associate degree	66 (10.8)
Bachelor's degree	156 (25.4)
Master's degree	89 (14.5)
Advanced degree	36 (5.87)
Employment status	
Not retired	369 (60.2)
Retired	244 (39.8)

Abbreviation: GED, general educational development.

### Prevalence of Accommodation Requests and Job Loss

**Figure 1:**
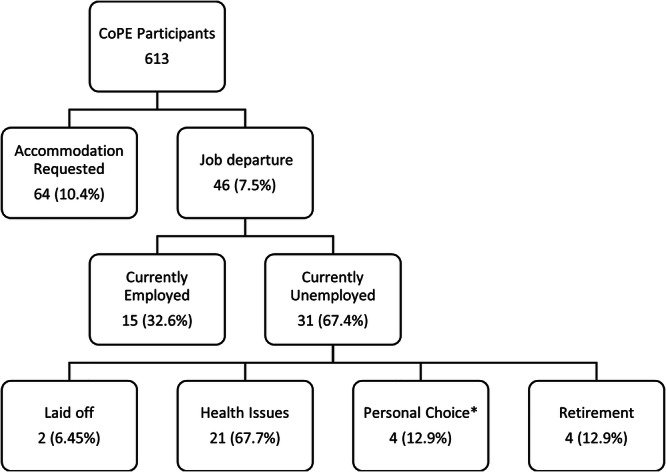
Flow of participants. *Personal choice includes choosing not to be employed, having family responsibilities such as caring for children or other family members, or engaging in volunteer activities. CoPE, Cord Paralysis Experience.

Among the 613 participants, 64 (10%) individuals requested job accommodations, and 46 (7.5%) reported job loss due to their UVFP. Of those that had left a job, 31 (67%) remained unemployed at the time of the survey. Within that subset (n = 31), health issues were the most cited reason for unemployment (n = 21; 68%) (Figure 1). Of the 64 participants who indicated that they requested accommodations, 44 (69%) recorded their occupation. Those that requested accommodations tended to work in education, healthcare, or sales, whereas those who had experienced job loss more often worked in healthcare (Table 2).

**Table 2:** Occupations of Cord Paralysis Experience Participants Who Had Requested Accommodations or Had Job Departure Following Unilateral Vocal Fold Paralysis

	Accommodation request	Job departure
	N = 44	N = 15
Standard occupation classification (SOC) major occupation groups	No. (%)	No. (%)
11‐0000 management	5 (11.3)	1 (6.67)
13‐0000 business and financial operations	4 (9.09)	1 (6.67)
15‐0000 computer and mathematical	1 (2.23)	‐
17‐0000 architecture and engineering	1 (2.23)	‐
19‐0000 life, physical, and social science	‐	‐
21‐0000 community and social service	1 (2.23)	‐
23‐0000 legal	1 (2.23)	‐
25‐0000 education, training, and library	8 (18.1)	1 (6.67)
27‐0000 arts, design, entertainment, sports, and media	1 (2.23)	1 (6.67)
30‐0000 healthcare support, practitioners, and technical^b^	8 (18.1)	7 (46.7)
33‐0000 protective service	‐	‐
35‐0000 food preparation and service related	‐	‐
39‐0000 personal care and services	‐	1 (6.67)
41‐0000 sales and related	7 (15.9)	1 (6.67)
43‐0000 office and administrative support	4 (9.09)	1 (6.67)
47‐0000 construction and extraction	1 (2.23)	‐
49‐0000 installation, maintenance, repair	‐	‐
51‐0000 production	‐	‐
53‐0000 transportation and material moving	‐	1 (6.67)

^a^ Only professions involved in this study were reported. For a full list, please refer to Mori et al.^20^

^b^ Combination of two major occupation groups “29‐0000 healthcare practitioners and technical” and “31‐0000 healthcare support.”

### Factors Associated With Accommodation Requests

Multivariate logistic regression analysis found that being a woman and having more education were associated with a higher likelihood of requesting job accommodations related to UVFP (Table 3). In fact, women were 2.6‐times more likely to ask for accommodations than men (OR 2.6, 95% CI: 1.4‐5.0, *P* = .01). Participants with education beyond high school were much more likely to ask for job accommodations than those who only finished high school or earned a general educational development (GED) (OR 4.5, 95% CI: 1.4‐14.7, *P* = .01). Etiology was associated with requesting accommodation. Specifically, work accommodation was requested by 14% of patients whose UVFP etiology was surgery compared to 7.8% among other etiologies (*P* = .02). Otherwise, we found no association between etiology of UVFP and accommodation requests.

**Table 3:** Summary of Univariate and Multivariate Analysis of Variables Associated Accommodation Requests

	Unadjusted UVFP accommodation (beta, 95% confidence interval)	*P*‐value	Adjusted UVFP accommodation (beta, 95% confidence interval)	*P*‐value
Intercept	−2.73 (0.88)	.002	−4.20 (0.65)	<.0001
Age	.99 (0.97, 1.01)	.14	‐	‐
Women	3.00 (1.50, 6.00)	.002	2.60 (1.35, 5.0)	.004
Black	1.87 (0.47, 7.50)	.38	‐	‐
White	1.03 (0.32, 3.31)	.97	‐	‐
Other	2.47 (0.53, 11.43)	.25	‐	‐
Associates degree	.40 (0.14, 1.19)	.10	‐	‐
HS or GED	.24 (0.07, 0.80)	.02	4.52 (1.39, 14.74)	.01
$75,000‐$99,000 per year	.41 (0.15, 1.10)	.08	‐	‐

Abbreviations: GED, general educational development; HS, high school; UVFP, unilateral vocal fold paralysis.

### Factors Associated With Job Loss

Participants who requested accommodations more often had experienced job loss due to their condition (26.1% vs 9.2%, *P* < .01). Participants who made less than $14,000 annually made up a larger proportion of individuals who lost their job due to UVFP, than those who did not lose their job (19.6% vs 7.2%, *P* = .01) (Table 4). In multivariate logistic regression, those who had requested prior accommodations for their UVFP had 4.5‐fold increased odds of experiencing UVFP‐related job loss (OR 4.5, 95% CI: 2.1‐9.7, *P* < .01). Participants who were unpaid at the time of the survey due to illness had 6.4 increased odds of experiencing UVFP‐related job loss (OR 6.4, 95% CI: 3.3, 12.4, *P* < .01) (Table 5). We found no association between UVFP etiology and job loss.

**Table 4:** Comparison of Participants With and Without Job Loss

	No job loss N = 567	Job loss N = 46	*P*‐value
Age, mean (SD)	58.0 (15.4)	55.4 (12.5)	.27
Sex			
Women	63.7	73.9	.17
Men	36.2	26.1	.17
Race			
White	82.6	82.6	.99
Asian	3.9	0.0	.40^b^
Black	7.6	10.9	.42
Native Hawaiian/other Pacific Islander	0.4	0.0	1.0^b^
American Indian/Alaska Native	0.7	0.0	1.0^b^
Other or unspecified	3.5	6.5	.24^b^
Ethnicity			
Hispanic	6.5	13.0	.1
Employment status			
Currently employed for pay	43.7	32.6	.14
Retired	32.0	8.7	<.01^b^
Unemployed due to illness	13.4	45.7	<.01
Requested accommodations	9.2	26.1	<.01
Education			
Educational attainment, mean (SD)	4.9 (1.9)	4.8 (1.9)	.76
Some high school	2.3	4.3	.31^b^
High school/GED	17.1	13.0	.48
Associates	10.4	15.2	.31
Trade school	3.5	4.4	.68^b^
Some college	20.3	23.9	.55
Bachelors	25.9	19.6	.34
Masters	14.6	13.0	.77
Advanced degree	5.8	6.5	.74^b^
Income			
<$14,000	7.2	19.6	<.01
$15,000‐$24,000	4.9	8.7	.29^b^
$25,000‐$34,000	5.9	2.2	.50^b^
$35,000‐$49,000	10.7	15.2	.35
$50,000‐$74,000	18.1	15.2	.62
$75,000‐$99,000	13.9	15.2	.81
$100,000‐$149,000	16.9	15.2	.77
$150,000‐$199,000	9.2	6.5	.79^b^
$200,000+	9.3	2.2	.17^b^

Abbreviation: GED, general educational development.

^a^ Data are presented as % unless otherwise indicated.

^b^ Indicates Fisher's exact test was used.

**Table 5:** Summary of Univariate and Multivariate Analysis of Variables Associated With Job Departure

	Unadjusted job loss (beta, 95% confidence interval)	*P*‐value	Adjusted UVFP job loss (beta, 95% confidence interval)	*P*‐value
Intercept	−3.73 (0.44)	<.0001	−3.12 (0.24)	<.001
Women	1.44 (0.70, 2.96)	.33	‐	‐
Hispanic	1.5 (0.50, 4.5)	.47	‐	‐
Race other	1.48 (0.33, 6.53)	.61	‐	‐
Paid work	1.40 (0.53, 3.34)	.49	‐	‐
Unpaid: illness	6.65 (2.84, 15.6)	<.01	6.43 (3.3, 12.4)	<.0001
Unpaid: volunteer	16.2 (1.25, 209.8)	.03	13.7 (1.2, 158.6)	.04
Accommodation	4.10 (1.83, 9.20)	<.01	4.5 (2.1, 9.7)	.0001
<$14,000 per year	1.59 (0.65, 3.88)	.31	‐	‐
$200,000+ per year	0.24 (0.03, 1.86)	.17	‐	‐

Abbreviation: UVFP, unilateral vocal fold paralysis.

## Discussion

Our multicenter CoPE collaborative study found that people with UVFP had a substantial employment burden. Specifically, 1 in 10 affected participants had requested job accommodations, and 1 in 13 experienced job loss related to their condition. Estimating 20,000 individuals are diagnosed with UVFP annually in the United States,^2^ our findings would translate into more than 2000 people requesting job accommodations and 1600 experiencing job loss due to this condition per year. These are likely significant underestimates since we only captured the treatment‐seeking population.

This study contributes to understanding the patient‐level burden on people's ability to perform workplace duties, its impact on job security, and UVFP's societal impact on the workforce. Our results highlight the need to efficiently rehabilitate UVFP patients to reduce quality of life consequences and workplace burden. These results also highlight the importance of clinicians understanding that patients with UVFP qualify for and often require job accommodations and are at risk of job loss.

To our knowledge, only two single‐institution case series have evaluated the effects of UVFP on occupation, finding that 25% and 41% of patients made job changes due to their UVFP.^12^, ^13^ Our CoPE cohort was composed of 613 people with UVFP from 34 institutions across the United States and recorded types of jobs worked by participants and measured the prevalence of accommodation requests and job loss related to their condition. In comparison to prior studies, we found that 10% of participants had requested job accommodations, and 7.5% suffered job loss because of their voice disability.

### Job Accommodations

In the United States, it is estimated that 25% to 30% of the population rely on their voice as the primary tool for their occupation.^7^ However, these national estimates derive from studies conducted in the 1990s and may not be reflective of the voice demands in the present workforce. Losing the ability to communicate effectively is a patient‐related factor that has major ramifications on one's work productivity and effectiveness. The ADAAA includes “speaking and communication issues” as a disability,^8^ which means that individuals with voice disorders have legal standing to request job accommodations. A recent study characterized four categories of facilitators and barriers that affected the likelihood a person would request job accommodation: (1) employee‐related factors, (2) accommodation‐related factors, (3) job‐related factors, and (4) social workplace‐related factors; the most important employee‐related factor was the affected person's knowledge that they qualify for job accommodations and their degree of self‐advocacy.^23^ All of these factors have relevance for patients with UVFP. Differences in awareness and self‐advocacy may contribute to disparities in accommodation requests for UVFP by gender and education as discussed below.

#### Gender and Job Accommodations

Women were more likely to request job accommodations. Reasons are multifactorial, but it is hypothesized to be driven by two primary features: (1) women tend to use healthcare at a much higher rate than men, especially in the working age demographic, and (2) women tend to be overrepresented in occupations that have higher vocal demands. Based on Medical Expenditure Panel Survey data, women use healthcare at a 30% to 40% higher rate than men, which provides them more opportunity to self‐advocate and for clinicians to educate them about their candidacy for job accommodations.^24^ In contrast, working‐age men use less preventative healthcare services and do not seek immediate treatment for many health problems, and this likely includes dysphonia.

Gender occupation propensity is another important factor in the higher rate of work accommodation requests among women. In our cohort, the most commonly held jobs were in management, education, healthcare, business and financial operations, and office and administrative support (Supplemental Table S1, available online). These are fields where women predominate. Specifically, the Bureau of Labor Statistics reports that women account for more than half of all workers within several industry sections with high vocal demands including education and health services (74%), financial activities (52%), and leisure and hospitality (51%). In contrast, men are overrepresented in less vocally demanding occupations like construction (89%), transportation/utilities (75%), agriculture (72%), and manufacturing (71%).^25^

#### Education and Work Accommodations

Education was also associated with requesting job accommodation. Consistent with prior studies,^26^ we found that the likelihood of requesting accommodations was lowest among respondents whose highest educational attainment was GED/high school, whereas individuals with more education were more likely to request accommodations.^26^ Reasons for these differences may relate to higher degrees of self‐advocacy in requesting accommodations and knowledge of the ADAAA among those with higher levels of education. It is also possible that individuals with higher education are more often in higher professional positions in the workplace making it easier for them to initiate and implement accommodations.^27^

Dysphonia from UVFP differs from other medical conditions that traditionally are equated with disability in the workplace (eg, needing wheelchair access). Moreover, dysphonia may not fit within the legal definition of disability in terms of “duration of impairment,” rendering the need for job accommodations inconsistent.^28^ Thus, many individuals with UVFP may not realize their dysphonia is classified as a disability under the law, entitling them to job accommodations and time off to pursue medical treatment. It is also true that not all workplaces are mandated to provide accommodations. In particular, businesses that employ less than 15 people do not need to adhere to the ADAAA policies.^8^ These smaller businesses have a higher percentage of employees with lower education levels,^29^ which may provide less educated individuals with less access to job accommodations. This could partly explain why accommodations were associated with educational attainment in our study.

### Job Loss

In our cohort, 7.5% of participants reported job loss related to their condition. Participants were more likely to lose their jobs if they had previously requested accommodation. Those that requested work accommodations were aware that their vocal disability limited their ability to do their job. Accommodations are designed to allow people to work with disabilities. The finding that those that were accommodated were more likely to experience job loss may suggest that accommodations made for the voice disability were inadequate. The survey was not designed to distinguish whether the participant or their employer initiated the job loss. Nonetheless, the fact that 1 in 13 participants lost their job despite treatments existing to rehabilitate their injury suggests that either our treatments are inadequate to normalize patients' ability to communicate or that patients did not have access to quality care. Evidence exists that current treatments may not be as effective as we believe. For example, the 1992 study by Gray et al found that 25% of patients who underwent definitive type I thyroplasty to improve their voice still needed to adjust their employment to accommodate their voice disability. It is likely that treatment outcomes have improved in the 30 years since that study, but our results show that a large proportion of patients with UVFP still ultimately experience job loss due to their disability, despite the availability of treatment. Voice‐reliant jobs may change over time with the advent of and advancement of artificial intelligence (AI) capable of generating human‐like languages. For example, many jobs have been or may be replaced by AI in the years to come in sectors like telemarketing, education, healthcare, administration, and finance. These changes may impact the workforce's reliance on voice and may allow for patients with voice disorders like UVFP to contribute in other ways that are less voice‐intensive. This evolving technological landscape represents an additional factor that could influence employment outcomes for patients with UVFP.

## Limitations

Survey studies can only report on questions that were asked. In this study, we did not ask about the type of accommodations that participants with UVFP requested or the circumstances around the job loss related to UVFP. This is important information that would provide greater context for the results reported herein. Thus, we have to make suppositions about these issues, which will only be answerable by further exploration. Our survey did ask participants to report any comorbidities such as chronic health conditions (eg, cardiovascular disease, malignancy, rheumatologic diseases, kidney disease, diabetes, pulmonary diseases, and dysphagia). Although we recognize the importance of understanding how comorbid conditions influence employment outcomes, the focus of this study was on accommodations and job loss directly related to UVFP. A third limitation relates to generalizability, as it is possible that included participants did not accurately represent the overall population of patients with UVFP. Every effort was made to reduce this likelihood by recruiting and incentivizing participants from across the United States to complete the survey. These concerns are further assuaged by the fact that the sociodemographic characteristics of our participants are similar to those reported in other large, published national cohort studies of voice disorders.^30^, ^31^, ^32^, ^33^ A fourth limitation is that our study did not evaluate the effectiveness of surgical and nonsurgical rehabilitative treatments. Finally, the survey offered only cross‐sectional data, which allowed us to know whether a participant had undergone prior treatment, but did not inquire whether the job accommodation or job loss occurred before or after treatment, or the effect that treatment had on accommodations or job loss.

## Conclusion

To our knowledge, this is the largest study to date that has evaluated the occupational impact of individuals with UVFP. We found that UVFP has major impacts on employment with 1 in 10 of those affected requesting job accommodations and 1 in 13 experiencing job loss. Our results highlight the importance of timely, effective treatment for UVFP to reduce voice disability and the need for treating physicians to provide support for patients who may require job accommodations due to this condition.

## Author Contributions

**Koffi L. Lakpa**, Study design; data acquisition; manuscript drafting; manuscript approval; agreeable to all aspects of work. **Andrew Bowen**, Study design; data acquisition; manuscript drafting; manuscript approval; agreeable to all aspects of work. **Ezra Menon**, Study design; data acquisition; manuscript drafting; manuscript approval; agreeable to all aspects of work. **Sydney Ring**, Study design; data acquisition; manuscript drafting; manuscript approval; agreeable to all aspects of work. **Peter Nordby**, Data acquisition; manuscript drafting; manuscript approval; agreeable to all aspects of work. **Miranda Rasmussen**, Data acquisition; manuscript drafting; manuscript approval; agreeable to all aspects of work. **Natalia Arroyo**, Study design; data acquisition; manuscript drafting; manuscript approval; agreeable to all aspects of work. **Jiwei Zhao**, Study design; data acquisition; manuscript drafting; manuscript approval; agreeable to all aspects of work. **Sara Fernandes‐Taylor**, Study design; data acquisition; manuscript drafting; manuscript approval; agreeable to all aspects of work. **David O. Francis**, Study design; data acquisition; manuscript drafting; manuscript approval; agreeable to all aspects of work. All listed persons meet criteria for authorship, having contributed to the study design, data acquisition, manuscript drafting, manuscript approval, and agreeing to be accountable for all aspects of work.

## Disclosures

### Competing interests

There are no conflicts of interest to report.

### Funding source

This research and David O. Francis are supported by funding from the National Institute on Deafness and Other Communication Disorders, National Institutes of Health; 5K23DC013559‐07 (PI: David O. Francis); R21DC016724 (PI: David O. Francis).

## Supporting information

Supporting Information.

Supplemental Table S2: CoPE Collaborative author list. Description: Complete list of providers, by medical center, who enrolled patients in this study and are members of the CoPE Collaborative.
