# Supplementary material for: Job Accommodations and Job Loss From Unilateral Vocal Fold Paralysis
Source: Otolaryngol Head Neck Surg. 2025 Jun 12;173(4):938–46. doi: 10.1002/ohn.1334 (PMC12278813; doi:10.1002/ohn.1334)
Supplement: Supplementary file 1 — Supporting Information. [file OHN-173-938-s002.docx]

| **Supporting Table 1:** Occupation of CoPE participants who provided a job description at the time of the survey | |
| --- | --- |
| **Standard Occupation Classification (SOC) Major Occupation Groups** | **N=262***  No. (%) |
| 11-0000 Management | 30 (11.5) |
| 13-0000 Business and Financial Operations | 29 (11.1) |
| 15-0000 Computer and Mathematical | 7 (2.67) |
| 17-0000 Architecture and Engineering | 3 (1.14) |
| 19-0000 Life, Physical, and Social Science | 2 (0.76 |
| 21-0000 Community and Social Service | 16 (6.10) |
| 23-0000 Legal | 8 (3.05) |
| 25-0000 Education, Training, and Library | 30 (11.5) |
| 27-0000 Arts, Design, Entertainment, Sports, and Media | 18 (6.87) |
| 30-0000 Healthcare Support, Practitioners and Technical** | 37 (14.1) |
| 33-0000 Protective Service | 1 (0.38) |
| 35-0000 Food Preparation and Service Related | 5 (1.91) |
| 39-0000 Personal Care and Services | 6 (2.29) |
| 41-0000 Sales and Related | 42 (16) |
| 43-0000 Office and Administrative Support | 20 (7.63) |
| 47-0000 Construction and Extraction | 3 (1.14) |
| 49-0000 Installation, Maintenance, Repair | 6 (2.29) |
| 51-0000 Production | 1 (0.38) |
| 53-0000 Transportation and Material Moving | 13 (4.96) |
| *Only participants who listed a main occupation were included in the occupational classification system; **Combination of two major occupation groups “29-0000 healthcare practitioners and technical” and “31-0000 healthcare support” | |
